# Recent Trends and Innovation in Additive Manufacturing of Soft Functional Materials

**DOI:** 10.3390/ma14164521

**Published:** 2021-08-12

**Authors:** Jaime Eduardo Regis, Anabel Renteria, Samuel Ernesto Hall, Md Sahid Hassan, Cory Marquez, Yirong Lin

**Affiliations:** 1 Department of Mechanical Engineering, The University of Texas at El Paso, El Paso, TX 79968, USA; arenteriamarquez@miners.utep.edu (A.R.); sehallsanchez@miners.utep.edu (S.E.H.); mhassan2@miners.utep.edu (M.S.H.); cmarquez10@miners.utep.edu (C.M.); ylin3@utep.edu (Y.L.); 2 W.M. Keck Center for 3D Innovation, The University of Texas at El Paso, El Paso, TX 79968, USA

**Keywords:** additive manufacturing, soft materials, smart materials, stretchable devices

## Abstract

The growing demand for wearable devices, soft robotics, and tissue engineering in recent years has led to an increased effort in the field of soft materials. With the advent of personalized devices, the one-shape-fits-all manufacturing methods may soon no longer be the standard for the rapidly increasing market of soft devices. Recent findings have pushed technology and materials in the area of additive manufacturing (AM) as an alternative fabrication method for soft functional devices, taking geometrical designs and functionality to greater heights. For this reason, this review aims to highlights recent development and advances in AM processable soft materials with self-healing, shape memory, electronic, chromic or any combination of these functional properties. Furthermore, the influence of AM on the mechanical and physical properties on the functionality of these materials is expanded upon. Additionally, advances in soft devices in the fields of soft robotics, biomaterials, sensors, energy harvesters, and optoelectronics are discussed. Lastly, current challenges in AM for soft functional materials and future trends are discussed.

## 1. Introduction

Soft materials have developed as the key materials to address challenges in engineering fields where flexibility, large motions, and lightweight are desired. These types of materials can be easily deformed by thermal and mechanical stresses owing to their low Young’s modulus at room temperature (<100 MPa) [1] and high elongation without breaking. Additionally, soft materials can be found in various states such as colloids, liquids, gels, and colloids and polymers. Many soft materials display inherent structures or properties that can be significantly altered through external stimuli in a controlled manner and can be regarded as functional materials. Examples of these intrinsic functional properties include shape memory, dielectric, self-healing, and color-changing properties. Some soft materials may display more than one of these properties or can respond to multiple stimuli and are considered to be multifunctional. Additionally, soft material can be given magnetic, piezoelectric, or piezoresistive properties by incorporating functional filler. 

Additive manufacturing (AM) of soft materials has been gaining popularity in recent years due to the growing interest in wearable electronics, tailored biomedical implants, and soft robotics. In the rapidly increasing market for soft devices, the one-shape-fits-all approach may soon no longer be the standard. Because of the limitations and cost associated with creating new geometries through traditional manufacturing methods, AM has been considered the future for soft material processing. In AM, a computer designed structure is sliced into individual layers and then these layers are physically realized through a variety of methods such as deposition of build material through a nozzle, jetting of binder onto a build material substrate, photopolymerization of a material vat, or thermal coalescence of powder particles. AM techniques enable freedom of geometrical design, customization with no added cost, and fabrication of complex geometries. 

Materials that are otherwise rigid can be engineered to achieve softness and stretchability through advanced geometries and then be considered engineering soft materials [2]. Advanced geometries such as lattice and auxetic structures have previously been used to tune the mechanical properties of polymer and polymer composites which has allowed for some rigid polymers to have larger deformations without rupture. Examples of these are seen in many biomedical applications where material compliance to the geometry of the human body is necessary. The geometric freedom granted through AM has led to progress in these advanced designs; for this reason, engineering soft materials will be considered in this review.

The many AM technologies that have been developed have aided in the development of functional materials through intelligent design of structures, the development of stimuli-responsive 3D geometries, and functionality gradients through multi-material use in each individual layer. Next, different AM technologies and their application in soft functional materials manufacturing will be discussed. Then, the recent advancements, future trends and governing mechanism for AM processed functional materials will be detailed. Lastly, advances and challenges in applying these functional materials will be discussed. 

### 1.1. Material Extrusion

Material Extrusion (ME) is an AM method that selectively deposits material through a nozzle onto a movable substrate in a layer-by-layer fashion to produce a three-dimensional part. ME is the most commonly used methodology, especially for rapid prototyping due to low cost, ease of use, and market availability. The most two common techniques for ME are fused deposition modeling (FDM) and direct ink write (DIW) also known as robocasting, paste extrusion (PE) or bio-extrusion (when applied for biomedical purposes).

FDM and DIW differ in the materials they use, and the way solidification is achieved. FDM uses thermoplastic filaments and has a fast solidification process through the cooling of the printed material below its glass transition temperature [3]. DIW does not require the temperature to achieve solidification and instead works with feed materials that flow due to a shear-thinning effect and retain their shape after deposition thanks to a high storage modulus (G’).

FDM has been used to successfully fabricate soft functional devices using thermoplastic polyurethanes (TPU), and shape memory polymers (SMP). In contrast, since DIW only has the requirement of appropriate rheological properties for its print materials, it has expanded to include a large soft material selection including polymers with a wide range of molecular weights, liquid crystal elastomers hydrogels, and SMPs [4].

ME techniques are also of interest since they can incorporate multi-head nozzles to realize devices with elaborate designs with the use of selectively deposited support material or can produce devices with structural and functional regions by carefully placing different materials together using the different nozzles [5]. Common applications for these AM technologies have been sensors, soft robotics, biomedical, and wearable devices [6,7].

### 1.2. Vat Photopolymerization

Vat photopolymerization (VP) is an AM method in which a vat of liquid photopolymer resin is selectively cured by a light source in a layer-by-layer manner to construct a three-dimensional object. VP includes various techniques that differ in the way that the light source, typically ultraviolet (UV) light, is projected onto the liquid photopolymer reservoir [8]. In processes such as stereolithography (SLA), micro-stereolithography (u-SL), and two-photon polymerization (TPP), light is projected from a single-point source that traces the part’s shape until a full layer is built. Processes such as digital light processing (DLP) and continuous liquid interphase production (CLIP) operate with a specialized projector that cures an entire layer at once. 

VP is one of the more promising AM methods for soft material printing due to the ability to fabricate delicate objects with high resolution, close tolerances, and smooth surface finishes. Additionally, the ability to incorporate filler material into the resin allows for tunability of physical and mechanical properties leading to better control over the functionality of the final part. Despite the numerous advantages, there is a lack of available soft materials compatible with the technology. Moreover, end-use parts manufactured through VP often require more extensive post-processing steps than other AM methods and have limited production size and strength, narrowing the use of the technology for many practical applications.

VP has been previously used to print soft materials through free radical polymerization (FRP) by attaching acrylate and methacrylate functional groups to elastomer monomer, allowing for the formation of covalent crosslinks upon UV exposure. In recent years, however, research on soft material printing through VP has been moving towards click chemistries, especially thiol-ene reactions, due to the mild reaction conditions, insensitivity to oxygen or water, rapid polymerization rate, high efficiency, and low cytotoxicity. Thiol-ene click chemistries have become an efficient tool to covalently crosslink polysiloxanes into silicone elastomers, gels, and encapsulants, and have received great attention in 3D printing technologies since it exhibits high resolution and accuracy through instantaneous formations of crosslinked networks only at the local and temporal exposure to UV-radiation [9]. 

### 1.3. Material Jetting

Material jetting (MJ) is an AM method in which droplets of liquid photopolymer are selectively deposited and cured successively layer by layer. MJ offers fewer manufacturing difficulties than other AM methods such as vat polymerization, which ensures similar resolution between prints and a higher rate of production. MJ also provides a more efficient method of deposition, line-wise deposition, compared to the other AM methods previously discussed in which deposition is point-wise. Thus, the technology can achieve high accuracy and smooth surface finishes often without the need for post-processing.

MJ consists of several techniques, including drop-on-demand (DOD), PolyJet printing, and nanoparticle jetting (NPJ). DOD printers operate by accurately depositing photopolymer resins on a substrate and subsequently curing through UV-radiation layer by layer until the full structure is created. In PolyJet printing, an ultra-thin layer of photopolymer resin is sprayed on the build platform and cured through UV light. Gel-like support materials that can easily be removed by hand or dissolved are used in this technique to support complex geometries. Lastly, NPJ nanoparticles or support nanoparticles are incorporated in a resin that is sprayed onto a build platform in the form of tiny droplets. Solvents used for flow in the nanoparticle resin are then evaporated by the high temperatures inside of the machine, leaving behind structures made from nanoparticle materials. 

MJ systems have become popular in recent years due to their capability for multi-material printing through DOD [10]. Although used for more advanced purposes, the MJ printing methods share many similarities to traditional 2D document printing systems making it possible for 3D printers to easily be used in an ordinary office rather than a laboratory environment. Advances in MJ technology have led to the fabrication of technology such as heat-responsive active composite structures [11] and functionally graded actuators for soft robots [12].

Although MJ is a promising AM method for soft materials, the technology, just like other AM methods, suffers from limited availability of materials that are printable. Additionally, wax-like materials, which are widely used due to their compatibility with MJ tend to be rather fragile, limiting the application for the structure produced through this method.

### 1.4. Other Additive Manufacturing Methods

Table 1 summarizes the AM methods previously discussed. The remaining AM methods (binder jetting, directed energy deposition, and sheet lamination) will not be discussed in further detail since these methods are typically reserved for processing metals, ceramics, or hard polymers and are not used for the development of soft structures (inherent and engineered) as previously described. 

## 2. Materials and Methods

### 2.1. Shape Memory Polymers

Shape memory polymers (SPs) are stimulus-responsive materials that can store different geometries in memory as a “temporary shape” and then return to their permanent shape by applying an external stimulus. SMPs have a high entropy state at their permanent shape, however, when stress is applied and reaches plastic deformation at their transition temperature (T_trans_), this allows them to reach a low entropy state. When cooling to temperatures below their T_trans_ without removing the stress, the entropy is frozen at a low state, which allows a temporary shape to be fixed by trapping the kinetic energy [22,23]. Lastly, when a stimulus is applied at T_trans_, the chain mobility of the material is reactivated and returned to its high entropy state [22,23]. SMPs must have a soft and a hard component, forming an interlinked polymer chain to activate functionalization of the material with a reversible shape memory effect (SME). The soft component allows elastic deformations or the shape morphing at T_trans_, while the hard component, usually a crosslinker, determines the permanent shape of the material. The performance of the SME for shape-memory materials is typically characterized by measuring their shape fixity ratio (R_s_), which measures the material’s capability to be deformed into a temporary shape, and their shape recovery ratio (R_r_). Table 2 further summarizes AM processed SMPs, the AM method used for their fabrication, glass transition temperature, elastic modulus, elongation at break, durability, shape fixity and shape recovery.

SMPs have recently gained significant attention due to diverse advantages such as their light weight, flexibility of programming mechanisms, high shape deformability, biocompatibility, and biodegradability for actuator applications. SMPs are also attractive materials to fabricate diverse types of sensors for soft robotics and aerospace applications and for minimally invasive surgery devices for biomedical purposes. 3D printing offers an effortless way to fabricate more elaborate designs, AM has the flexibility to control some of the factors that have been found to affect SME’s performance including geometry, print path direction, and thickness of the sample [33]. VP has been of interest for processing SMPs due to its in-situ polymerization process that allows the fabrication of elaborate geometries for very specific applications. SLA and DLP have been reported in the fabrication of origami structures, biomimetic, and soft robotic devices. Choong et al. evaluated the use of nanosilica dispersed in tBA-co-DEGDA photocurable resin using DLP to enhance nucleation and accelerate the polymerization rate, which significantly reduced fabrication time with Rs of 100% and Rr of 87% [24]. For ME, FDM and UV-assisted DIW have been reported in the fabrication of SMPs with biomedical purposes and soft robotics application [25]. Villacres et al. used the FDM technique to print a semi-crystalline TPU where they evaluated the effect of printing orientation and infill on the SME. It was found that a print angle orientation of 60° and 100% infill resulted in an increment of failure strain and strength where the infill content had a higher influence on mechanical properties [34]. Chen et al. fabricated tough epoxy and N-butyl Acrylate SMPs composites by UV-assisted DIW with Rs of 97.1% and Rr of 98.5% [26]. Lastly, Jeon et al. fabricated multicolored photo responsive SMP structures through Polyjet printing that showed different geometries when triggered by assorted color lights of different wavelengths [35]. 

There are two main classifications by stimuli response known as thermal-responsive and chemo-responsive. Thermal-responsive SMPs are triggered by applying heat to the material and raising their temperature up to their Ttrans. However, using a direct heating method could restrict their applications, which has led to the use of functional fillers to fabricate SMPs composites that trigger SME by alternative methods, such as electricity, magnetism, light, and ultrasound. Liu et al. fabricated multi-responsive SMPs using Polycyclooctene with boron nitride and multi-wall carbon nanotubes (MWCNTs) by the FDM technique. By using 20 wt.% MWCNTs in the composite, the SME could be triggered by heat (under water at 70 °C), light (100 mW·cm^2^), and electricity (5 V) with outstanding properties, Rs of 98.9% and Rr of 99.2% [27] Zhang et al. fabricated PLA-Fe_3_O_4_ composites by FDM using magnetism (27.5 kHz) as an alternative stimulus for SME. It was found that a higher content of Fe_3_O_4_ led to a higher Rr, where PLA-Fe_3_O_4_-20% mass fraction gave the best results with Rs of 96.8% and Rr of 96.3% [28]. 

In chemo-responsive SMPs, the SME is triggered by altering the ionic strength to promote plasticizing and lower Ttrans below room temperature [36]. The most common method consists of submerging the SMP in a medium, such as an organic solvent or water, that triggers the plasticizing. Recovery time can be decreased by reducing the dimensions of the polymers to micro-fibers [37]. Solvent-responsive SMPs commonly report the use of organic solvents such as ethanol, dimethyl sulfoxide methanol, and N-N dimethylformamide (DMF) [3, 12]. Some water responsive SMPs includes hydrogels (Polyvinyl alcohol, polyethylene glycol) [38] and TPUs [37]. Shiblee et al. fabricated water-responsive shape memory gels by SLA process using poly (dimethyl acrylamide-costearyl acrylate and/or lauryl acrylate) (PDMAAm-co-SA) by incorporating hydrophilic and hydrophobic monomers in the formulation. This shape-memory gel showed an Rs of 99.8% and Rr of 87.6% after the first cycle and Rr of 99.8% after the second cycle, the authors attributed the change of Rr to the training phenomenon [29]. 

Thermal-responsive SMPs are mainly activated by hot programming, which consists of heating the material to its T_trans_. The main advantage of hot programming is a high R_s_, a minimal springback and it usually requires a small amount of applied stress to produce a plastic deformation [39]. Li et al. fabricated Bisphenol-A glycerolate diacrylate (BPAGA) SMPs by DLP technique using hot programming method at glass transition temperature obtaining R_r_ of about 97% and R_s_ of 100% [40]. On the other hand, chemo responsive SMPs are activated by either hot or cold programming. Cold programming is possible below T_trans_ and usually occurs at room temperature. However, cold programming is usually more challenging since some thermosets are brittle at their rubbery point leading to possible fractures [39]. Keshavarzan et al. evaluated hot programming and cold programming methods for BCC and rhombic structures using 3DM-LED.W, which is a commercial SMP resin for DLP. It was found that cold programming is beneficial for higher energy absorption while hot programming obtained a higher shape fixity ratio, it was also noticed that rhombic structures have a better energy absorption and recovery due to higher strength and stiffness [32]. 

SMPs show different behaviors according to the number of geometries that can be stored in memory, which depends on the network elasticity of the material [36]. Lastly, multi-SMPs are materials that can learn more than three geometries additionally to their permanent shape. Peng et al. synthesized triple SMPs by using poly(ethylene glycol) dimethacrylate (PEGDMA), isobornyl acrylate and 2-ethylhexyl acrylate through DLP. The SMPs were able to store two different geometries in memory with an Rs of 92.6% and Rr of 95.3% without a significant degradation after 10 cycles, proving the effectiveness of SMPs [41]. 

SMPs with chemical crosslinking are usually thermosets and have stronger bonds than physically crosslinked polymers and higher shape recovery. However, SMPs with chemical crosslinks cannot be reprocessed unless they have dynamic bonds. Some SMPs with chemical crosslinking take advantage of dynamic chemistries such as transesterification, transcarbamoylation, Diels–Alder bonds, disulfide bonds, diselenide bonds, and imine bonds [38]. Thermadapts are a type of SMPs with dynamic covalent bonds that have recently gained attention due to their capability to change the temporary shape after curing. Some of the dynamic covalent bonds used to fabricate SMPs are hindered urea bonds and triazolinedione. Miao et al. developed thermadapt SMPs (2-Methacryloyloxy and 4-formylbenzoate) with dynamic imine covalent bonds using DLP that allowed changing the temporary shape after printing for different actuation purposes that can be useful for soft robotics applications [30]. Davidson et al. used LCEs to develop thermadapt SMPs by radical-mediated dynamic covalent bonds using the hot DIW technique. When exposed to UV light during actuation, the exchangeable bonds that allow the change of the permanent shape of LCE are activated [42]. Some SMPs that reported the use of physical crosslinking include hydrogen bonds, ionic bonds, π-stacking, charge transfer interactions [38]. Chen et al. synthetized PET copolyester using π-stacking synergistic crosslinking to induce enhance shape memory properties by the FDM technique. The optimal copolyester was P(ET-co-PN) 20 with an Rs of 100% and Rr of 98%, it was also found to have some levels of self-healing due to π-stacking crosslinking and flame retardant properties due to the nature of PET [31]. 

SMPs have a diverse range of applications due to their unique mechanism, where 3D printing contributes to the evolution of elaborate designs. Many efforts to control the responsiveness by alternative methods besides direct heating have been made. An interesting research direction could be the development of SMPs with dual responsive mechanisms for different purposes that expand their fields of application. Furthermore, multi-material printing may allow the fabrication of SMPs that can store multiple geometries in memory. The evaluation of 3D printing structures to fabricate reprocessable SMPs with dynamic covalent bonds is another interesting research direction that can redefine SMPs’ functionality.

### 2.2. Self-Healing Materials

Self-healing polymers are a branch of functional materials designed to take advantage of intricate physical or chemical processes to reform broken bonds caused by mechanical damage. The ability of self-healing polymers to respond to damage that may be difficult to detect, helps prevent the propagation of cracks or ruptures that result from the polymer’s exposure to fatigue, abrasion, and other deteriorating forces during regular operation. Recent advances in AM have led to an increase in the development of self-healing materials that overcome the design limitations of traditional casting methods, resulting in self-healable structures with increased complexity and tunable properties. For this reason, the application of self-healing polymers has extended beyond protective coatings to wearable devices, implantable biomedical devices, health monitors, and electronic skins.

The healing efficiency of AM processed self-healing polymers is often measured through the restoration percentage of physical properties such as fracture strain and corresponding tensile or compressive stress. Additionally, the recovery time can also be an indicator of performance and in the case of non-autonomic processes, the activation energy required to trigger the self-healing process, which can be calculated through the Arrhenius equation. Table 3 further summarizes AM processed self-healing polymers, the AM method used for their fabrication, their functional chemistry, recovery performance, recovery conditions, and applications.

In the past two years, there has been a surge in the AM of crosslinked gels with reversible imine Schaff bonding. For example, Kim et al. demonstrated the design and preparation of biocompatible, polysaccharides-based, self-healing hydrogels [53]. These hydrogels were processed through extrusion-based bioprinting to form stable geometries such as donuts, disks, and filamentous structures. The hydrogels displayed autonomic healing ability in ambient conditions (room temperature in air). The healing was based on reversible crosslinks comprising of imine bonds and hydrazine bonds, that were capable of completely restoring functionality within 10 min. The same research group took the concept one step further by incorporating iron oxide nanoparticles. This ferrogel was capable of fully recovering autonomously after gel breakage in a lapse of 10 min in three different conditions (in air at room temperature, in a buffer solution, and under a magnetic field). Additionally, this ferrogel also demonstrated shape memory capabilities that were triggered by the presence of a magnetic field [44]. In another example, Lei et al. synthesized a gelatin-based self-healing hydrogel from dialdehyde carboxymethyl cellulose and amino-modified gelatin. The hydrogel showed good fatigue resistance by recovering its original strength during 10 cyclic compressive loading and unloading tests and by having a healing efficiency of up to 90% after being heated for 1 h at 37 °C. Additionally, the hydrogel possessed ideal hemocompatibility and cytocompatibility, making it a prospective candidate for injectable tissue engineering scaffolds [54].

While self-healing gels are mainly processed through material extrusion AM techniques, most self-healing elastomers are processed through UV-based methods such as VP and MJ. Due to the nature of the technology and the rigidity of elastomers compared to gels, AM processed self-healing elastomers can obtain more complex designs, higher resolutions, better surface quality, and overall faster printing speeds, especially through the use of click chemistries such as thiol-ene photolymerization. 

Pertaining to VP, Liu et al. demonstrated the fabrication of hydrolysis-resistant silicone elastomers through photopolymerization conversion of vinyl in thiol-ene photoreactions in a stereolithography process [49]. This self-healing silicone elastomer demonstrated a healing efficiency higher than 90% when healed at 100 °C for 12 h. The silicone elastomers could be healed multiple times through reversible ionic crosslinks without losing significant strength. Furthermore, the silicone elastomers were shown to be reprocessable, retaining 85% of their original strength when pulverized and re-casted. Similarly, Yu et al. developed a photocurable PDMS-based elastomer through the same photopolymerization and AM technique but with dynamic covalent crosslinks [46]. Through disulfide exchange, this photoelastomer was able to completely regain 100% of its original strength in significantly less time (2 h) and under milder conditions (60 °C) than the silicone elastomer Liu et al. prepared, however, with less translucency. 

In regard to UV-light-assisted methods, Kuang et al. developed a novel semi-interpenetrating polymer (semi-IPN) composite for UV-light-assisted DIW that displayed self-healing properties [43]. Photocurable resin composed of urethane diacrylate, and n-butyl acrylate was incorporated to assist in the shape retention of the printed beads of the material when post-cured after extrusion of every layer. This allowed the group to fabricate complex structures with high stretchability like an Archimedean spiral capable of stretching over 300% strain with negligible in-plane anisotropy. The healing in the semi-IPN elastomer is based mainly on the diffusion of a semicrystalline polymer, polycaprolactone, and partially from hydrogen bonding between urethanes. As such, the elastomer demonstrated the ability to heal micro-cracks (3 mm long and 30 µm wide) after heat treatment at 80 °C for 20 min leaving only slight scarring. The elastomer was also able to heal larger cracks such as a notched gap, however, the healing efficiency was relatively low (<30%) in comparison to the self-healing materials previously discussed. Additionally, the elastomer possessed shape memory capabilities associated to the crystalline component of the elastomer. While self-healing elastomers still have a long way to go to be ready for consumer-based applications, in terms of mechanical robustness, significant progress has been made over the past few years to achieve faster healing under mild conditions (low temperatures/pressure) and better printability. 

Despite self-healing materials being some of the most varied, AM processable self-healing polymers are still relatively limited due to the novelty of AM technology. One of the shortcomings of self-healing polymers is their fragility, especially for gels. Through free-standing AM techniques such as DIW and MJ, the hydrogels are susceptible to collapsing under their own weight. Although the use of support particulate gel beds is not uncommon, the manufacturing process of the particle can significantly affect the quality of the gel. For this reason, Senios et al. developed a fluid-gel bed that provides support to 3D extruded structures and prevents them from collapsing under their own weight prior to being crosslinked [55]. This process, known as suspended layer additive manufacturing (SLAM), was shown to be able to overcome limitations associated with printing low viscosity inks such as spread when depositing and sagging in multilayered structures, thus allowing for the fabrication of hydrogels with even more intricate designs than previously achievable. The development of AM assisting techniques such as SLAM, will help broaden the material catalog and allow for their use in a wider range of applications and push for commercial goods such as wearable electronics. 

### 2.3. Electronic Polymers

Electronic soft polymers are those that exhibit changes to their electronic properties, such as polarization, capacitance, or resistance when exposed to mechanical, thermal, light, or pH stimulus. These characteristics have made electroactive polymers useful for actuating [56], sensing [57], and energy harvesting [58] applications, and to develop as a very important research area. 

There exist various families of electronic polymers defined by the governing physical mechanism of their functionality. Examples of electronic polymers include dielectric elastomers, piezoresistive polymers, and piezoelectric polymers. These polymer families have been explored for AM to produce conformable, smart structures with programmed sensing and actuating behaviors. 

Dielectric elastomers (DEs) are electroded elastomers that respond with large actuation to applied electric fields due to their high compliances. The Coulombic forces that result from the applied electric field cause a reduction in the thickness and anisotropic expansion of the electrode area of the elastomer. DEs are quite useful as actuators for biomedical [59] and soft robotics [56] because of their large strains, low noise, and quick response times. However, DEs have inherent disadvantages such as requiring large electric fields upwards of 100 kV/mm [60] and having isotropic non-directed deformations. 

AM processes have been used in recent years to obtain three-dimensional dielectric elastomer actuators (DEAs). AM provides advantages to DEs such as the ability to build different elements such as the DE films, the electrodes, or the rigid frames together using a wide variety of deposition methods. ME and MJ technologies have been mainly used for the manufacturing of DEs [61], Progress in MJ of DEAs has been driven by the development of rubbers suitable for jetting into films with controlled distribution and thickness. For example, AM patterned films were obtained through MJ using commercial silicone rubber by diluting them in a solvent to obtain suitable jetting properties prior to printing [61]. Careful design of the printing inks resulted in prints with comparable properties to traditionally casted films. Another approach to building DEAs using MJ that has been explored is the aerosol jetting of graphene oxide electrodes onto DE films [62]. The capability to pattern electrodes onto dielectric elastomer films allowed for electrode patterns that were unaffected by substrate stretching and it was proposed that stacked DEAs could be produced by alternating layers of silicone and graphene oxide jetting.

Multi-material manufacturing approaches enabled by AM have been used to fabricate fully functional DE actuators. In one example, a unimorph cantilever was built by selective deposition of an active barium titanate/silicon DE layer, a passive stiff silicon layer, and ionogel electrodes [63]. All different elements were built using a single AM process and the resulting cantilever bent upon the application of an electric field. In a similar multi-material approach, 3D actuators were manufactured by printing rigid thermoplastic frames on a prestretched acrylic DE substrate using FDM [64]. Stretched DEs were used as a substrate so that once the print was finished and the substrate was released, the contraction of the substrate would transform into bending due to the mismatch of modulus across the thickness. The resulting curled-up structures followed predictions using minimization of energy approach and showcased how three-dimensional complex actuators could be built using intelligent design of printed patterns onto a DE substrate. Similarly, honeycombs of TPU were patterned using FDM into both sides of acrylic DE substrates to obtain anisotropic unidirectional actuators [65]. Higher degrees of anisotropy were obtained by varying the rib angle of the honeycombs and increasing the pre-stretch ratios of the DEs during print. Under optimal conditions, an axial strain of 15.8% with only −0.97% transverse strain was achieved by loading with 7.5 kV. 

Overall, the necessity to pre-stretch DEs to obtain useful actuation behaviors limits the incorporation of these materials into all AM processes. DEs will see further development only in multi-material approaches where complete actuators can be built in one AM process. Still, smart design for AM will continue to develop and result in actuators with efficient electromechanical energy transfer.

Piezoresistive materials respond with a change in electrical resistance when strained. In the case of piezoresistive polymer composites, a network of electrically conductive fillers embedded in a thermoplastic or thermoset elastomer matrix is disturbed by strain causing a variation in the electrical conductivity of the composites [66]. This variation in electrical resistance as a function of strain if large, can be used to accurately measure strain. For piezoresistive polymer composites, sensitivity is maximum when the concentration of the filler approaches what is known as the percolation threshold. At this concentration, it is possible for agglomerations to form and impact the sensitivity. Thus, manufacturing methods for piezoresistive composites must ensure that agglomerations do not occur. Among the suitable manufacturing processes for piezoresistive composites, AM has emerged as one of the most important prospects because of the freedom in design, and because of the control of filler alignment possible in processes such as ME. 

Piezoresistive soft polymer composites have been manufactured through ME using different matrix and filler systems [67,68,69,70,71]. For example, sensors of TPU with CNT fillers were manufactured using FDM [68]. The use of AM enabled new or enhanced properties in some cases. For example, biaxial strain sensors made of TPU with CNT fillers were manufactured using FDM [68]. The different patterns of CNT electrode deposition allowed for different designs, each with its own sensitivities to axial and transverse deformations, with largely unaffected mechanical properties (all materials exhibited ≈50% axial strain at 4 MPa loading). In another work, a hierarchically porous lattice of TPU was printed and bonded to a stretchable matrix of the same TPU and used as a conformable sensor [70]. The macroscale porosity was controlled by the spacing of the struts, while intermediate and small-scale porosities were achieved through sacrificial fillers burned out after printing. The hierarchical porosity achieved anisotropy in response allowing the sensor to be bonded to curved substrates without affecting its pressure sensitivity. This was not previously possible for conventionally casted sensors. Other approaches used in AM of piezoresistive polymer composites to enhance their performance have consisted of using particle–matrix interface modifiers [67] and embedding CNTs to a printed elastomer lattice using partial melting [69].

Piezoresistive soft polymer composites have been manufactured through ME using different matrix and filler systems [67,68,69,70,71]. The use of AM enabled new or enhanced properties in some cases. For example, biaxial strain sensors made of TPU with CNT fillers were manufactured using FDM [68]. The different patterns of CNT electrode deposition allowed for different designs, each with its own sensitivities to axial and transverse deformations, with largely unaffected mechanical properties (all materials exhibited ≈50% axial strain at 4 MPa loading). In another work, a hierarchically porous lattice of TPU was printed and bonded to a stretchable matrix of the same TPU and used as a conformable sensor [70]. The macroscale porosity was controlled by the spacing of the struts, while intermediate and small-scale porosities were achieved through sacrificial fillers burned out after printing. The hierarchical porosity achieved anisotropy in response allowing the sensor to be bonded to curved substrates without affecting its pressure sensitivity. This was not previously possible for conventionally casted sensors. Other approaches used in AM of piezoresistive polymer composites to enhance their performance have consisted of using particle–matrix interface modifiers [67] and embedding CNTs to a printed elastomer lattice using partial melting [69]. Ionic gels are another class of piezoresistive materials that have been developed for AM. A group of researchers developed a shear-thinning ionic gel that could be patterned into 3D structures and studied how a reentrant honeycomb structure enabled 310% larger elongations and sensitivity as compared to a traditional film [72]. Another group further developed ionic gels for printing using eutectic solvents as the media for better stability post-printing [73]. Once again, the freedom of design from AM was used to construct auxetic structures that offered enhanced strain sensitivity with a max GF of 3.30 and a strain of 300%. 

Piezoresistive polymers have been widely developed for AM using ME. Further control of the porosity, and design of metamaterial structures will enable enhanced sensitivity and a broader range of operation. However, other AM processes with higher resolutions such as VP will be needed to develop smaller piezoresistive polymer sensors for use in MEMS.

Piezoelectric materials possess a permanent polarization that when disturbed through mechanical loading produces a voltage across the material. These materials are electromechanically coupled so mechanical loads produce voltages and applied voltages cause strains. Because of this characteristic, piezoelectric materials can function as both electrically driven actuators [74] and as mechanical sensors [75]. 

Flexible piezoelectric materials have been developed with the help of AM by printing polymers as well as nanocomposites with piezoelectric ceramic fillers. Optimization of concentration, along with intelligent structure design, has allowed materials to exhibit larger coupling coefficients as well as increased elongations. Strategies for AM of soft piezoelectrics have focused on intelligent structure design to overcome the inherent stiffness of common piezoelectric materials such as PVDF and ceramics. For example, Li et al. used electrical field-assisted FDM to produce piezoelectric sheets with designed deformations made with nanocomposites of sodium niobate ceramics and PVDF [76]. Chiral patterns were built into the sheets to allow large deformations. Thus, once the sheets were rolled onto artery-like structures they could expand to sense radial pressures such as those found in blood flow inside the human body. Similarly, Yao et al. developed flexible and wearable piezoelectric sensors using lattice patterns through DLP [77]. Highly sensitive but soft piezoelectric lattices were possible thanks to surface functionalization of the piezoelectric ceramic fillers, which enhanced mechanical energy transfer at lower solid loadings. Three-dimensional honeycomb structures were printed using the high-resolution photopolymerization printing method and the performance of the sensors surpassed piezoelectric polymers in sensitivity and compliance. Another approach towards building compliant piezoelectric structures through AM consisted of infiltration of a ceramic lattice with PDMS elastomer [78]. This strategy allowed for complex structures to be built using only a ceramic-filled resin using SLA while still being able to obtain complaint structures afterwards.

Soft piezoelectric materials have been developed for AM despite the limited selection of polymer systems that exhibit piezoelectric polymers and the high stiffness of bulk nanocomposites of piezoelectric ceramics and elastomers. Continued development of metamaterial structures and identification of unique piezoelectric behaviors will continue to drive AM of soft piezoelectric structures composed of polymers and ceramic fillers.

Table 4 below summarizes the different efforts to print electronic polymers for sensing and actuation applications, highlights the specific elements fabricated using AM, their softness, and their individual performance.

### 2.4. Chromic Materials

Chromic materials have the ability to change in appearance in their refractive index (e.g., color, fluorescence, brightness, transparency) when applying different stimuli such as temperature, mechanical stress, electricity, pH concentration, among others. Chromic materials are of interest due to their reversible optical mechanism that can be incorporated into wearable devices for sensing, and for soft robotics. Table 5 further summarizes AM processed SMPs, the AM method used for their fabrication, applied stimuli, color change, absorbance wavelength and reversibility.

The use of AM for soft chromic materials provides an effortless method to explore different geometrical designs and obtain tunable, mechanically activated chromic (also known as mechanochromic) responses with reversible optical properties. For example, Rohde et al. used the DIW technique to explore different geometries for mechanochromic composite elastomers, using PDMS microbeads as a matrix with spiropyran aggregates as a functional filler [80]. Activation of the chromic mechanism was possible by applying either a low mechanical strain under uniaxial tension or compression. The soft elastomer composite showed reversible mechanochromic properties displaying a purple color in the area of applied mechanical force and returning to white after releasing such force. Chen et al. fabricated highly stretchable photonic crystal hydrogels with reversible mechanochromic properties by DIW technique [81]. The physically crosslinked poly(butylacrylate) (PBA) composites provided a high elongation at break of 2800% and reversible color change from blue to grey under tension and compression. 

Additionally, AM has contributed to the fabrication of complex inks with moisture-activated chromic properties (often referred to as hydrochromism) that allow the obtainment of multiple color changes. For example, Yao et al. developed a 3D printable hydrogel ink for the DIW technique to develop soft actuators with shape memory and appearance tuning properties [85]. By using polyethyleneimine-co-poly (acrylic acid) (PEI-co-PAA), hydrochromism was produced, showing tunable luminescence from blue to green, which can be controlled by water absorption of the actuators in the range of 20% to 90% relative humidity. Moreover, by the incorporation of fluorophore-lanthanide an additional red color was also tunable by water absorption in the sample. The hydrochromic soft actuators also showed a reversible change in opacity from opaque to transparent produced by phase separation caused by dehydration. 

Thermally activated chromic (often referred to as thermochromic) are the most reported chromic materials due to the simplicity of their chromism mechanism tuning. One example is Chen et al. who developed a 3D printable resin with polyurethane acrylate (PUAs) oligomer and isobornyl acrylate (IBOA) monomer with shape memory properties for the µSL process [82]. By the addition of thermochromic microcapsules, it was possible to fabricate self-actuating devices with reversible change colors from red to white with tunable glass transition temperature from 74.2 to 81.7 °C. 

Electrically activated chromic materials (also known as electrochromic) have been of interest for soft functional devices as an alternative method to used temperature to trigger a color change. For example, Zhou et al. used DIW technique to fabricate a device using poly(N-isopropylacrylamide) (PNIPAm) as functional particles dispersed in an Si/Al sol-gel, producing a hybrid hydrogel (PSAHH) with reversible appearance properties from opaque to translucent that could be triggered by heat or electricity [83]. These reversible thermochromic and electrochromic properties could be triggered by heating the samples above 60 °C or by increasing current from 0.6 to 1.8 Amp (2~6 V). The change in the sample’s appearance was due to the temperature-induced dehydration of PNIPAm particles, which acted as light scattering fillers. Cai et al. used ink-jet printing to fabricate electrochromic WO3-PEDOT:PSS composites printed on flexible substrates with electrochromic properties [84]. The flexible device showed a fast electrochromic response even under bending conditions in the range of −0.6 to 0 V transitioning from transparent to black and with good electrochemical stability up to 10,000 cycles. 

Chromic materials are an emerging research area that has recently found its way into AM techniques for the fabrication of soft functional devices. Due to the high potential of chromic to fabricate wearable devices, it is expected to see future research trends taking advantage of AM to develop multi-responsive devices with reversible chromic properties.

### 2.5. Multifunctional Soft Materials

Multifunctional materials are those that present two or more functionalities due to their inherent properties or when combined with other functional materials as composites. The functionalities that make up multifunctional materials can be a combination of shape memory, self-healing, actuation, sensing, optical, biological, elastic, etc. In engineered multifunctional material systems, properties are carefully selected to achieve the desired multifunctionalities based on the field of applications. For example, multifunctional biomaterials must first present a therapeutic functionality and then may present added functionality including sensing of body temperature and pressure, or actuation [86]. Other engineered multifunctional materials can provide structural support in demanding environments while providing additional functionality to address very strict requirements [87]. The applications of such materials include energy [88], medicine [87], nanoelectronics, aerospace, defense, semiconductor, and other industries.

Multifunctional materials reduce system complexity by having one material perform functions that would be otherwise performed by multiple different materials. This is beneficial in applications such as soft robotics where weight reduction and simplicity are some of the key characteristics and the use of the least number of materials ensures the best performance possible [89]. The integration of multifunctional materials into structures requires material compatibility and adhesion between different components. AM allows a seamless transition from structural to functional sections through material gradients [90]. Thus, the combined development of materials with multiple functionalities together with the development of AM techniques that easily transition between materials allows for the simplest, most size effective structures. Table 6 below summarizes the different multifunctional materials and composites that have recently been developed using a variety of AM processes.

One of the families of advanced soft materials with the greatest potential for multifunctionality enabled through AM is hydrogels. These materials possess molecular networks swollen in water that, when subject to stimuli such as temperature or strain, may undergo gelling. Gelling constitutes a physical change where the stiffness of the hydrogel, as well as other relevant properties such as the swelling behavior, are altered. Thus, these materials possess sensing and shape change capabilities. Additionally, due to their high inherent compliance, they offer the potential to form functional composites with metallic or carbon fillers without sacrificing their softness. Functional hydrogel composites with electrically [93] or magnetically [31,71,72] responsive fillers have been enabled through AM. For example, hydrogels with self-healing molecular networks have been combined with conductive carbon fillers and used to fabricate complex sensing and healing structures through DIW [93]. Similarly, self-healing hydrogels with magnetic iron particle fillers were printed using DIW [44]. The structures could heal damage over time through reversible imine-bond formations, and the printed structures also exhibited macroscopic shape change in the presence of a magnetic field. Additionally, hydrogels were printed simply through DIW with the use of carbomer as a rheological modifier and their multifunctional properties were showcased [91]. The printed hydrogels showed time-dependent actuation in a hot (50 °C) water environment due to a phase transition and deswelling of one of the printed layers at the water bath temperature. The showcased hydrogels were also mixed with magnetic particle fillers and printed to form a biologically inspired octopus structure. This structure could locomote in the presence of a moving magnetic field and demonstrated the potential to obtain soft robotic nature mimicking structures through DIW [91]. Though magnetic hydrogel composites are capable of motion when a magnetic field is applied, their magnetic response is still considered weak. Thus, Tang et al. instead obtained 3D printed actuating structures that worked through the magnetothermal effect by introducing an alterning magnetic field causing heat and degradation of the hydrogel networks [95]. The printed parts were able to both encase and kill cancer cells after an oscillating magnetic field was applied due to the actuation of the heated hydrogel-filled elastomeric arms.

Composites with SMPs enabled through multi-material AM have shown potential to develop into multifunctional actuators. Bodkhe and Ermanni designed a piezoelectric SMP that changed its shape with temperature and could simultaneously measure the extent of its deformation through the development of a proportional voltage signal [92]. The multifunctional composite was enabled through the deposition of rigid and soft sections with multi-material DIW. The possibilities to tune actuation temperatures from 100 °C down to body temperatures were explored, and a robust sensor capable of withstanding temperatures ranging from 23 °C to 100 °C was presented, and to over 5000 operation cycles. This shape-memory composite had a shape recovery rate of ∼98% and the sensor had a linear voltage response in the force range of 0.1–1 N [92]. Ge et al. combined hydrogels and SMPs through a multi-material DLP print method. UV curable hydrogels were developed by the creation of a water-soluble photoinitiator [96]. To obtain heterogeneous structures of elastomer and hydrogel, a moving stage with “puddles” of the different precursor solutions was placed under the UV light at different times per layer and air-jetted off in-between material exchanges. The multi-material structures were able to perform multiple functions owing to the combination of advanced materials. For example, stents were built that could be cold programmed to a small diameter size and inserted into blood vessels and later would expand through the body temperature induced shape recovery process. 

AM has the potential to provide additional functionality through careful geometrical design. As an example, multifunctional silicone structures with hierarchical porosities were built using a combination of micrometer sized sacrificial pore forming fillers and macroscopic infill gaps through DIW [94]. The 3D printed structures that had millimeter sized gaps on their infill attained super hydrophobicity and super olephilicity due to their capacity to entrap air at the pores inside and around the printed struts. Moreover, the silicone inks used to print the porous structures were able to be mixed with CNTs, showcasing their capabilities as resistive sensors. The additional functionality of these structures as sensors was demonstrated by wetting them in a CNT bath, subjecting them to cyclic compressions, and measuring the linear electrical resistance response. A linear response was observed up to 10% strain although the highly porous structures could easily be compressed cyclically without loss of elasticity. 

3D printed multifunctional materials and structures will continue to develop through the development of AM compatible chemistries, as well as the incorporation of multi-material printing to the different AM technologies. Careful design of new materials will aim to achieve multifunctionality without compromising AM compatibility. The incorporation of heterogeneous materials into AM feed has been highly developed, and thus, the blueprints for a future of yet unrealized multifunctional composites are in place. Multifunctional materials may begin to outpace single function materials due to the benefits they provide including system simplicity and reduction of mass. However, careful design must ensure that coexisting functionalities are not compromised by the presence of one another. AM provides pathways to seamlessly transition from structural to functional elements, and the high resolution of various printing technologies will enable careful control of material deposition to ensure functionalities are not compromised. Together, the fields of AM and advanced materials will continue to develop hand in hand to realize a future with efficient, responsive, and intelligent structures for various industries. 

## 3. Applications

### 3.1. Biomaterials

Bioprinting has seen a surge in recent years helping progress the efforts in tissue and organ engineering through the incorporation of live cells into 3D printable inks that are engineered to interact with biological systems. Recent advancements in biological inks either promote the growth and interaction of cells and tissues or by becoming part of the cellular structure. In a recent example, Zhange et al. developed bioinks with the same biological activity as original cartilage extracellular matrices, which could be used to construct scaffolds for cartilage tissue engineering [97]. In another example, Butler et al. developed a bioink from a blend of chitosan and starch [98]. This bioink showed great printability and biocompatibility with great promise for neural tissue engineering applications.

Traditional methods for fabricating biomaterials such as casting, suffer from significant drawbacks such as suboptimal engraftment, poor cell survivability (~1%) and uncontrolled differentiation which limit their use in in vivo applications such as stem-cell therapy [99]. The ability to design and control the internal structure of biomaterials through AM has recently been seen as a method to overcome some of these barriers. Microarchitectural control in biomaterials, enabled through AM, can lead to better control of drug delivery and antibacterial properties of the material resulting in fewer infections and a lower likelihood of rejection by the immune system [100]. For example, Tytgat et al. reported on the potential of norbornene-functionalized gelatin and thiolated gelatin processed through the ME technique with a cell viability above 90% up to 14 days [101]. 

**Figure 1:**
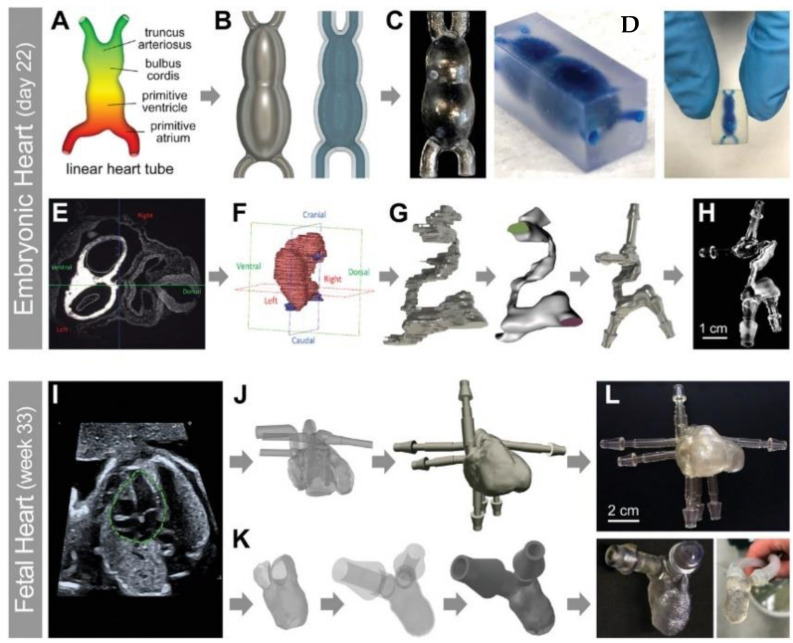
“Experimental workflow used to create patient-derived 3D printed heart models at varying developmental stages. A 3D model of an embryonic human heart at Carnegie stage 10 (21–23 days old, e-HT) was made either through (**A**–**D**) CAD modeling of an idealized structure, or (**E**–**H**) using the actual human data obtained in collaboration with the 3D Atlas of Human Embryology. The anatomical heart tube model contained the (**E**) primitive cardiovascular system which was (**F**–**H**) trimmed to only include the linear heart structure. (**I**–**L**) A patient-specific fetal left ventricle (f-LV) was acquired from fetal echocardiography of the human heart (week 33). The (**I**) full heart model was (**J**–**L**) trimmed at the mitral and aortic valves to only include the inner surface of the LV. For both models, the geometries were optimized, hollowed, and smoothed using the Meshmixer. Flow extensions were appended using AutoDesk Fusion 360 at the trimmed inlets and outlets to simulate adjacent vasculature. Different scales of (**C**,**H**) e-HT and (**L**) f-LV constructs were 3D printed by a Form 2 printer using the clear resin.”—[A. D. Cetnar et al., “Patient-Specific 3D Bioprinted Models of Developing Human Heart,” Adv. Healthc. Mater., vol. n/a, no. n/a, p. 2001169. Reproduced with permission.].

Bioprinting has primarily been used to produce higher quality models of tissue and organ to study complex diseases and anomalies such as cancer in vitro. For example, Zhu et al. bioprinted prevascularized tissues with complex 3D microarchitectures without the need of sacrificial material [102]. The use of bioprinting allowed the group to study different architectural features of the vascular network to produce a pre-vascularized tissue with high cell viability in vitro and in vivo. In another example, Mollica et al. developed a 3D biomimetic in vitro model through ME technique, using extracellular matrix from decellularized rat and human breast tissues [103]. This model was capable of sustaining large structural growths such as bioprinted organoid and tumaroid formations, or formations resembling organs and tumors. Another group from Georgia Institute of Technology and Emory University demonstrated the potential of producing anatomically correct, live, and functional organs through patient-derived models of the human heart at different stages of development through DLP of gelatin methacrylate bioinks (Figure 1) [104]. The development of advanced models can help in the discovery of diseases and treatments as well as significantly improve the reliability of those laboratory discoveries. In addition, the use of accurate and functional tissue and organ models could lead to reduced reliance on animal models.

**Figure 2:**
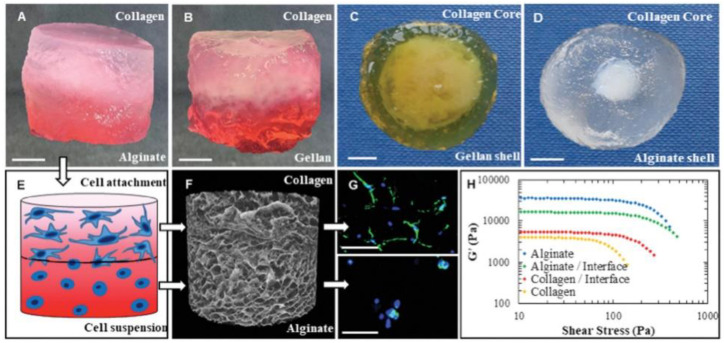
“3D printing multilayer gradient scaffolds by SLAM. (**A**,**B**) Images of bilayer scaffolds using combinations of (**A**) collagen–alginate and (**B**) collagen–gellan. (**C**) Large collagen–core gellan–shell scaffold and (**D**) small collagen–core alginate–shell scaffold (scale bars = 5 mm). (**E**) Schematic of diagram showing control of cell behavior with attachment motif bearing complexes in the upper collagen gel and no attachment motifs for cell suspension within an alginate gel. (**F**) Micro-CT showing gradient porosity within a lyophilized collagen–alginate scaffold. (**G**) Confocal micrographs of Hoechst/actin cell staining of HDFs attached in the collagen layer (upper) and suspended in the alginate (lower) regions of a dual layer scaffold (scale bars = 100 µm). (**H**) Stress versus G′ showing variations in gel strength and elasticity across a collagen–alginate scaffold including the interfacial regions illustrating a gradient in mechanical strength across the printed part.” [J. J. Senior, M. E. Cooke, L. M. Grover, and A. M. Smith, “Fabrication of Complex Hydrogel Structures Using Suspended Layer Additive Manufacturing (SLAM),” Adv. Funct. Mater., vol. 29, no. 49, p. 1904845, 2019. Reproduced with permission.].

One of the challenges in fabricating scaffolds for soft tissue and organs through AM is the lack of available biomaterials with the necessary mechanical and physical properties to be compatible with AM technology [105]. Most common biomaterials such as collagen, gelatin, fibrin, chitosan, hyaluronic acid, and silk suffer from poor mechanical strength which results in melting, dissolution and warping of the printed structure [106]. Recent approaches to better the rheological properties of bioinks have been seen through the incorporation of polymer microspheres [107]. Additionally, advances in the AM technology such as the development of Suspended Layer Additive Manufacturing (SLAM) for ME-based bioprinting have helped to achieve a higher complexity in the printed structures as shown by Figure 2 [55].

### 3.2. Sensors

Precise sensing is critical for various applications including biomedical, structural health monitoring, aerospace, and chemical. Various conditions of interest to be sensed include strain, pressure, temperature, and the presence of a gas or liquid. Soft materials that develop electric responses to these stimuli are of interest because their high stretchability makes them suitable for attachment onto various types of surfaces, including human bodies as wearable sensors and electronic skins [108]. Conventionally manufactured soft sensors have been limited by their geometries and material arrangements. These types of sensors cannot fully explore the capabilities of their constituent sensing materials. For example, it has been shown that enhanced stretchability and sensitivity could be achieved with the use of designed cellular structures [109]. These kinds of structures are difficult to achieve using conventional manufacturing methods without the use of specialized tools.

AM techniques have been used as methods to develop both, casing and sensing elements, to build soft sensors with expanded material and geometric selection [110,111,112]. Gao et al. used a ME technique to create a substrate for a pressure sensor that had slots built into it where conductive elastomer composites were later cast [110]. This sensor was able to be attached to different objects with curved surfaces and could distinguish the different gripping forces exerted to lift objects. A similar approach was used by Zhou et al. who printed a piezoresistive strain sensor using SLA where the upper layer of the printed structure had channels that were filled with strain-sensitive Galinstan after the printing process [111]. The asymmetry of the printed structures enabled by the different patterns on each cured layer allowed for the determination of the type of bending motion experienced. 

Complete sensors have been completely built using AM taking advantage of the multi-material capabilities of processes such as ME, where multiple nozzles are filled with a unique material that can also be used in a single print [113]. The process of building a complete strain sensor using AM consisted of first depositing elastomer layers to act as insulators, then depositing electrode beads using a carbon nanotube-filled elastomer, and finally depositing a pressure-sensitive layer made from an ionic liquid-filled elastomer. The multi-material sensor performed reliably and the ability to deposit electrode traces in specific regions of the prior backing layers allowed for localization of pressure along the body of the sensor. 

**Figure 3:**
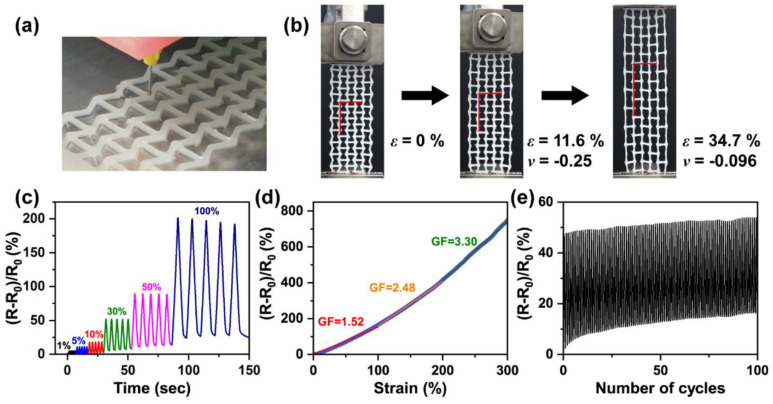
“Performance of 3D printed strain sensors with resistance change. (**a**) Photograph of the printing process. (**b**) Dimensional change in the auxetic sensor during stretching. (**c**) Relative resistance change in the sensor at different strains. (**d**) Variation in relative resistance vs. strain. The slope of linear fitting corresponds to the gauge factors in the range of strain from 0 to 100%, 100 to 200%, and 200 to 300%, respectively. (**e**) Relative resistance change in the sensor at 30% strain for 100 cycles.” [Chun-Wei LaiChun-Wei Lai, “3D Printable Strain Sensors from Deep Eutectic Solvents and Cellulose Nanocrystals|ACS Applied Materials and Interfaces.” https://pubs.acs.org/doi/10.1021/acsami.0c11152 (accessed 22 October 2020). Reproduced with permission.].

Various soft strain sensors have been recently built using a variety of AM techniques and materials [68,71,114,115,116,117,118,119,120]. Some of these developed sensors have shown enhanced out-of-plane sensitivity compared to casted sensors when 3D patterning using ME [117]. The use of print nozzles with a 100 μm diameter allowed for miniaturized flexible sensors that could sense very low pressures [117]. Different print patterns built using ME have also been used to develop strain sensors with augmented anisotropic strain sensitivities [68]. The capability to pattern complex structures out of sensing materials using AM was utilized by Lai and Yu who built wearable sensors with auxetic cellular geometries out of conductive hydrogels as shown in Figure 3 [73]. These sensors had vastly increased stretching limits when compared to solid geometries and could maintain contact with human skin even after muscle contractions and extensions. Yu et al. also demonstrated the advantage of building three-dimensional cellular geometries for strain sensors [121]. Lattices of thermoplastic polyurethane were built using FDM and then coated with carbon nanotubes to add strain-sensing functionality. The presence of carbon only on the surfaces as well as the inherent softness of the lattices resulted in very high sensitivity and stretchability. In a similar manner, Yin et al. used SLA to manufacture hydrogel electrodes with mesh geometries for capacitive strain sensors [122]. Their electrode designs enhanced the sensitivity of the sensors and they found that three-dimensional meshes exhibited higher strain sensitivities than two-dimensional meshes because of their higher compliance. 

**Figure 4:**
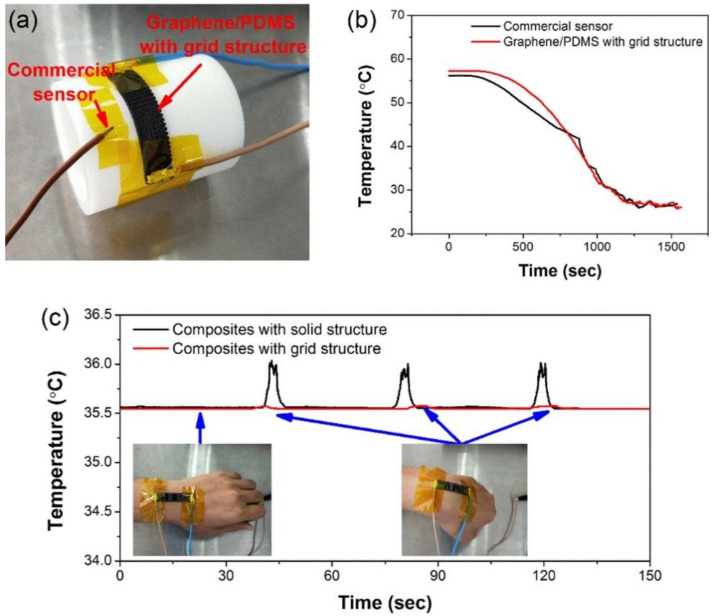
“Monitoring of the cooling process on a curved surface: (**a**) experimental setup; (**b**) comparison of the temperature performance between graphene/PDMS composites with a grid structure and commercial temperature sensor; and (**c**) simultaneous monitoring of wrist skin temperature and joint bending.” [Z. Wang et al., “3D-Printed Graphene/Polydimethylsiloxane Composites for Stretchable and Strain-Insensitive Temperature Sensors,” *ACS Appl. Mater. Interfaces*, vol. 11, no. 1, pp. 1344–1352, Jan. 2019.].

AM has enabled the development of soft sensors with enhanced and added functionalities. The capability to pattern unique cellular structures through AM was used to build a temperature sensor that was insensitive to strain, as shown by Figure 4 [123]. The use of cellular patterns built using ME allowed for the sensor to stretch without experiencing large amounts of elastic strain on the cellular trusses and thus reduced the sensitivity of its electrical properties to deformation. The built sensors had the ability to sense temperature in the same manner as undeformed sensors despite being bent and twisted to conform to curved surfaces. AM has also aided in the development of hydrogel-based liquid sensors capable of distinguishing the position and volume of the target liquid. Electrically conductive hydrogels were printed using a high-resolution multi-material SLA process [124]. By depositing sections of hydrogel with and without conductive carbon fillers, the researchers were able to build a mesh that could detect the position and volume of liquid in contact with the sensor. The different sensitivities of the neat and carbon-filled hydrogels allowed for bidirectional sensing of liquid coming from leakage in different sections of a system.

Soft sensors fabricated through AM have shown great potential as they allow for unique patterns with enhanced sensitivity to stimuli and unique multidirectional sensing. As new materials with better performance are developed for sensors, they will require manufacturing methods with great flexibility in the material selection that do not require complete retooling for the new materials. AM will continue to co-develop with emerging materials to result in highly sensitive and resistant sensors suitable for new operational environments. 

### 3.3. Energy Harvesting

Energy harvesters are devices that can generate electrical energy from alternative sources of energy, such as thermal and mechanical. Energy harvesting can take the form of triboelectric, thermoelectric, and piezoelectric devices, the last of which are among the most common [125]. With the push for sustainable green energy in recent years, the use of soft engineered materials for energy harvesting has gained significant interest due to their capability to conform to different surfaces, making them attractive in many modern fields such as automotive, aerospace, and biomedical applications. The use of AM technology has contributed to improved designs of energy harvesting devices by expanding the geometric selection to include lattice structures with better mechanical properties such as higher compression and conversion of energy, and easier incorporation towards their application [126]. With great shape conformity and elasticity, AM-enabled energy harvesters may find great use in applications such as wearable electronics, textiles, and skins [127,128].

**Figure 5:**
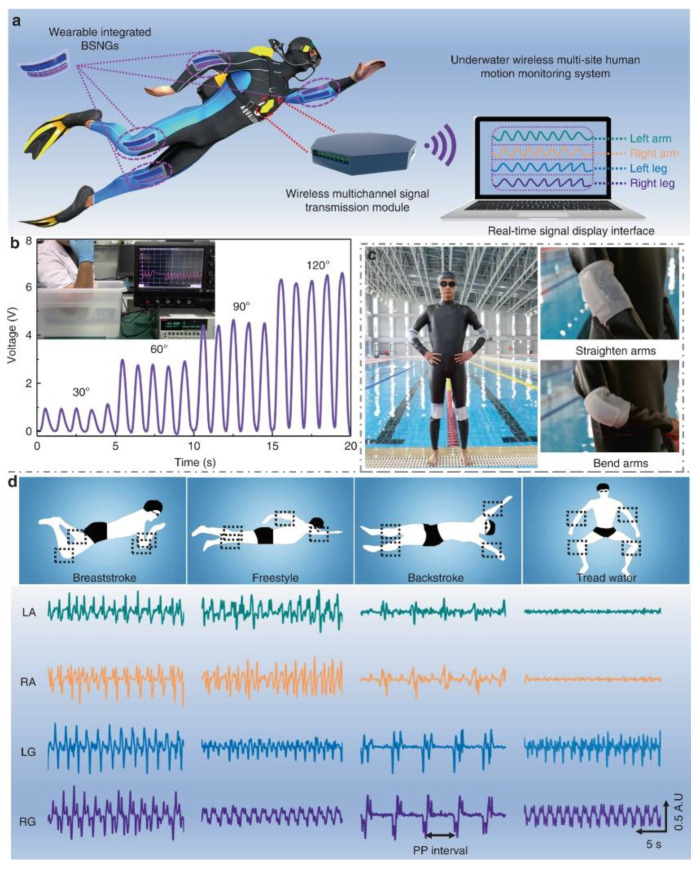
“Underwater wireless multi-site human motion monitoring system. (**a**) Illustration of underwater wireless multi-site human motion monitoring system based on bionic stretchable nanogenerator (BSNG). (**b**) Signal outputs of BSNG fixed on the elbow at different curvature motion. (**c**) Photographs of integrated wearable BSNG worn on the arthrosis of humans. (**d**) Signal outputs recorded by underwater wireless multi-site human motion monitoring system when the volunteer swam in different strokes (LA, RA, LG, RG represent left arm, right arm, left leg, right leg, respectively; PP interval represents the time interval between two peaks)” [Y. Zou et al., “A bionic stretchable nanogenerator for underwater sensing and energy harvesting,” *Nat. Commun.*, vol. 10, no. 1, p. 2695, Dec. 2019].

**Figure 6:**
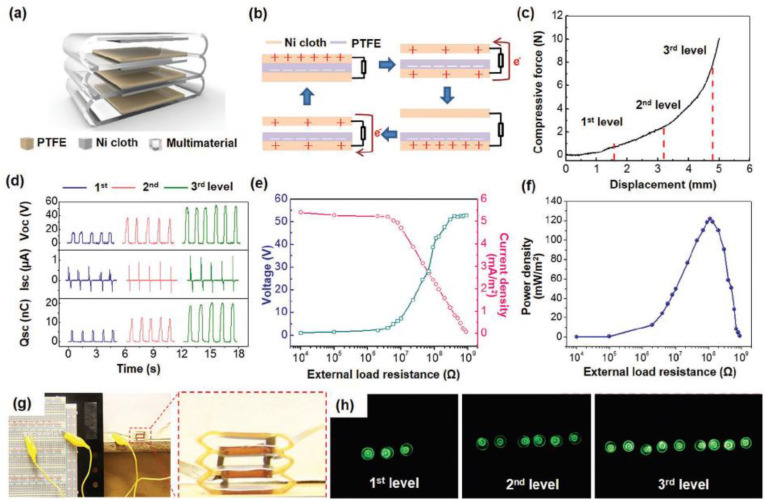
Design, working mechanism, and output performance of mTENG. (**a**) Schematic illustration of the 3D printing multi-material used as self-powered wearable electronics. (**b**) Schematic illustration of the electricity-generation process of the single-level contact separation. (**c**) Force–displacement curve of compressive test of the three-layer multi-material showing three stages of deformation. (**d**) The output performance of TENG in the different levels of contact-separation mode, including Voc, Isc, and Qsc. (**e**) Relation between the instantaneous output voltage and current density with the external load resistance. (**f**) Relation between the instantaneous output power density with the external load resistance. (**g**) The m-TENG was used as an energy harvester to lighten LEDs. (**h**) The mTENG can lighten 3, 6, and 9 LEDs by compression with the first, second, and third levels of deformation. [K. Chen et al., “Dynamic Photomask-Assisted Direct Ink Writing Multimaterial for Multilevel Triboelectric Nanogenerator,” *Adv. Funct. Mater.*, vol. 29, no. 33, p. 1903568, 2019, Copyright Wiley-VCH GmbH. Reproduced with permission.].

Various types of energy harvesters have been explored through AM using soft matrix materials that can respond to different stimuli such as motion, temperature, or stress. One type of energy harvester, the triboelectric device, generates electrical energy from materials that are electrically charged after they are separated from a different material. For example, Zou et al. fabricated a triboelectric device through DIW and were able to harvest the motion of muscles to power a sensor while underwater. This printed triboelectric device demonstrated good flexibility and stretchability for energy conversion while showing outstanding tensile fatigue resistance, as seen in Figure 5 [129]. In another example, Kaijuan et al. printed a triboelectric nanogenerator (TENG) energy harvester through DIW that could harvest mechanical energy from motion and power a sensor that was embedded in a shoe insole [130]. This sensor could be built into multilevel structures that could harvest energy due to the triboelectric effect of three sequential deformation stages as shown in Figure 6. The use of multi-material AM allowed for a single process programming of a TENG. Kaijuan et al. successfully provided a new approach to wearable devices by combining 3D printing with TENG devices for self-sustainable and comfortable embedded sensors. Another type of energy harvesting device is the thermoelectric device which can harvest energy from electric potentials generated from temperature differences across a distance Lazaros et al. printed an organic thermoelectric generator (TEG) with TPU and multi-walled carbon nanotubes through FDM for potential large-scale energy harvesting applications with stretchability [131]. One of the current challenges of thermoelectric energy harvesters is their limited use in industrial applications. However, the development of wearable devices where complex 3D architectures and customizability are required have opened new avenues for these harvesters. 

One of the most common types of energy harvesters is piezoelectric devices which are capable of transforming applied mechanical stress into an electric potential. As an example of the use of AM to better the properties of these devices, Xiaoting et al. used a combined DOD and ME printer to fabricate a multilayer piezoelectric energy harvester that could harvest higher amounts of power than conventional flat harvesters [132]. Annealing of the piezoelectric polymers after each layer was deposited allowed for a higher amount of ferroelectric phase development and an overall higher piezoelectric performance. The multilayer harvesters were wrapped around a “rugby ball” shaped part made of soft PDMS polymer that was printed using ME at a separate print process. Overall, the AM harvesters performed 2.2 times better in open circuit conditions compared to flat single layered harvesters and could develop an output power 100 times greater than flat, single-layered harvesters. In another example, Desheng et al. were also able to fabricate embedded piezoelectric sensors through µSL and obtained high piezoelectric responsiveness and compliance via exploiting the effects of nanoparticle–matrix functionalization of the electromechanical performance of the piezoelectric nanocomposite [77]. This allowed them to achieve target flexibilities while keeping high piezoelectric responses via rational designs of inclusion morphologies and monomer stiffness of the constituent materials. 

AM has greatly contributed to the development of energy harvesters by the incorporation of soft functional materials that can help to increase the range of application by allowing the adaptation to different surfaces and shapes. Furthermore, AM has contributed to being able to fabricate devices with a high degree of customization without design limitations, which has shown improved functionality for energy conversion. 

### 3.4. Soft Robotics

Soft robots and/or actuators are highly compliant devices with multiple degrees of freedom that enable object manipulation with minimal damage. Due to their interesting material properties such as flexibility and compliance, soft robotics have gained a significant attention in the fabrication of diverse types of grippers for manufacturing [133], nature inspired actuation [134,135], sea exploration [136], surgical devices [137,138], and rehabilitation devices [133,139]. Actuation of soft robots occurs usually through the application of pneumatic or hydraulic pressure [133], temperature [134,135,140], UV light [135,141], magnetic fields [134], or electric fields [137,138]. The most used materials for soft robotics applications include SMPs, hydrogels, ionic polymer–metal composites (IPMC), and elastomers. 

**Figure 7:**
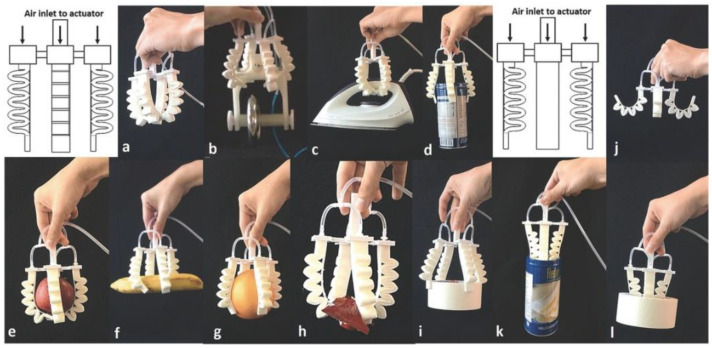
“Demonstration of (**a**) robotic gripper equipped with 3D-printed pneumatic actuators picking up (**b**) a 0.94 kg structure and (**c**–**j**) items of different shapes, sizes, and weight using 175 kPa of pressure. (**j**) A second robotic gripper with outward-facing 3D-printed pneumatic actuators that can grasp (**k**) inside of a cylindrical can and (**l**) roll of tape.” [B. A. W. Keong and R. Y. C. Hua, “A Novel Fold-Based Design Approach toward Printable Soft Robotics Using Flexible 3D Printing Materials,” *Adv. Mater. Technol.*, vol. 3, no. 2, p. 1700172, 2018, Copyright Wiley-VCH GmbH. Reproduced with permission.].

AM enables the tuning of mechanical properties of soft actuators by controlling the material orientation and chemical composition without design limitations and with outstanding performance. Some researchers have used AM to print molds and then traditionally cast a soft material with the purpose of being used as a soft actuator. However, this method has several limitations, such as limited structure design, poor mechanical properties, and extended manufacturing processes. AM used for direct printing of soft robotics devices is a cost-effective method that contributes to a reduction in the manufacturing lead time as well as facilitating the fabrication of custom designs. For example, Ang et al. fabricated different bellow-type of soft actuators (Figure 7) by FDM methods using NinjaFlex, which is a TPU-based filament, with the purpose of controlling bending and increasing length of actuation. At 175 kPa the bellow-type soft actuators could withstand a weight of 0.94 Kg and handle delicate objects such as food without damaging them [133]. Furthermore, Ang et al. demonstrated that besides being used as grippers for manufacturing, NinjaFlex soft actuators could also be used for locomotion, and wearable devices applications.

In addition, AM facilitates the engineering of mechanical properties to obtain fast actuation and control of bending curvature, which determines the efficiency of soft actuators. Electroactive hydrogels (EAH) for example, are materials of interest for soft actuators that exhibit a linear deformation dependence on an applied electric field. Han et al. used PμSL to control bending curvature and actuation of EAH by varying the thickness of several types of structures (grippers, transporters, and human-like) that enabled manipulation and locomotion due to their elaborate designs [141]. Saed et al. developed molecularly engineered LCEs to modify their thermomechanical properties and control their actuation temperature and strain. The soft actuators were fabricated by DIW with an extended actuation temperature range from 20 °C to 100 °C [140]. Roach et al. took advantage of the shear force generated on the ink during extrusion in DIW to align the liquid crystal monomers in the direction of printing and obtain large actuations. The alignment of monomers was locked as the material was deposited by shining a UV light and locking it through the formation of crosslinks. They demonstrated that printing with smaller nozzle diameters contributed to a higher actuation strain, additionally, the influence of printing speed on the alignment of LCEs was evaluated [142]. 

**Figure 8:**
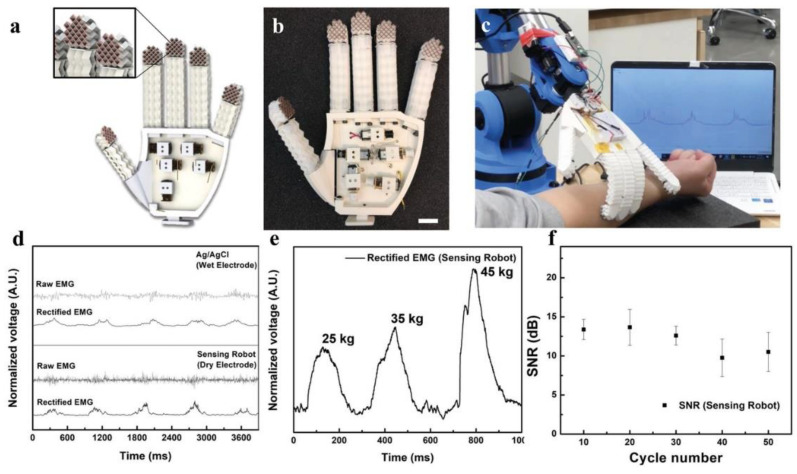
EMG assessment with origami-inspired robotic structures and materials. (**a**) Schematics and (**b**) actual photo image of electromyogram (EMG) sensing humanoid robot hand (scale bar: 2 cm). (**c**) Photo image of the sensing robot during the EMG measurement. (**d**) Typical raw and rectified EMG signals measured by wet Ag/AgCl electrodes (**top**) and the EMG sensing robot (**bottom**). (**e**) Rectified EMG signals at three different fist-holding forces of the participant. (**f**) SNR profiles of EMG sensing robot as a function of touching cycles to the human subject. [T.-H. Kim, J. Vanloo, and W. S. Kim, “3D Origami Sensing Robots for Cooperative Healthcare Monitoring,” Adv. Mater. Technol., vol. n/a, no. n/a, p. 2000938, Copyright Wiley-VCH GmbH. Reproduced with permission].

Soft polymer composites with functional nanofillers are another interesting approach for soft robots that either provides an alternative actuation mechanism or additional functionalities. Some researchers have used nanofillers to develop nature-inspired or mimetic soft actuators. For example, Kim et al. developed 3D origami soft sensing robots using FDM composed of nanocomposite filaments of cellulose nanofiber, PLA, and TPU, as shown in Figure 8. These devices could detect electromyography signals for health monitoring [143]. Tognato et al. created a method to tune the anisotropy of robotic actuation by adding magnetic responsive iron oxide nanoparticles (IOPs) to a PEG matrix suitable for AM. Due to the high biocompatibility of IOPs, this device could be used as a bioactuator for cell reorientation with a multi-stimuli responsive mechanism like starfish-inspired structures [134]. Finally, Kim et al. developed several conductive soft actuators by using MWCNT dispersed in epoxy aliphatic acrylate with tunable mechanical properties fabricated through DLP [144]. 

AM has shown great capabilities to expand the functionality and applications of soft robotics for manufacturing and biomedical purposes compared with traditional manufacturing methods. Some future trends in soft robotics might expand to include multi-material for single 3D printing steps of soft actuators and electrodes, improving the adhesion of materials and mechanical properties. Another interesting future approach is the evaluation of actuation mechanisms and tunable mechanical properties for metamaterial designs, which are only possible by AM techniques. In addition, some future trends for biomedical purposes include the fabrication of soft actuators for surgical devices, which might allow the development of body temperature responsive devices.

### 3.5. Optoelectronics

Optoelectronic devices place a connection between optics and electronics by generating light from electrical energy or producing energy by capturing light through a semiconductor. Optoelectronics can be applied as photodiodes [145], solar cells [146], or light-emitting diodes [147].

AM has drawn more attention in the manufacturing of optoelectronic devices over traditional microfabrication technologies in recent years. This is due to AM’s ability to extend the flexibility in the design and fabrication of 3D structured optoelectronics that results in high-performance integrated active electronic materials and devices. In addition, AM also allows for the integration of both, organic/inorganic/biological and conducting/semiconducting materials, as a single tool [148]. For example, Hu et al. [149] have shown black phosphorus as a functional ink platform for MJ of visible and near-infrared photoelectronic, including photodetectors. Additionally, AM’s ability to incorporate functional fillers has demonstrated tunable optical properties. A group from the University of Warwick utilized FDM to fabricate several electromagnetic devices capable of controlling the propagated wave through a particularly graded refractive index [150]. Such devices like 3D printed gradient refractive index lens showed ways to manipulate and control an electromagnetic wave going through a boundary between two homogeneous media. 

**Figure 9:**
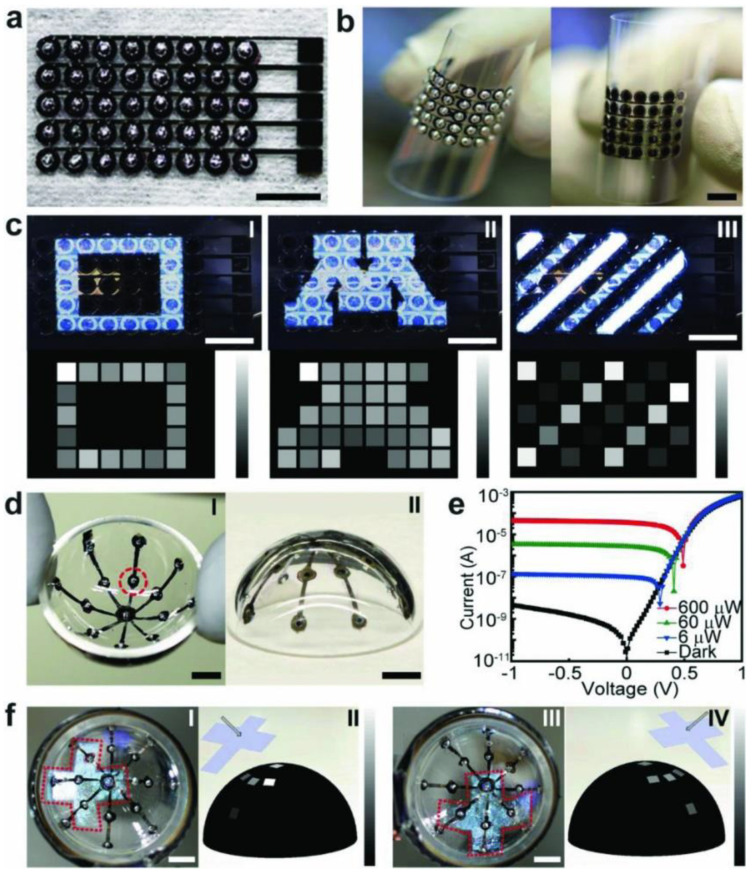
“3D-printed photodetector arrays printed on planar and spherical surfaces. (**a**) 5 × 8 photodetector array printed on PET. (**b**) Photographs of the bent photodetector array on PET films, viewed from both sides. (**c**) Characterization of the flexible photodetector array as an image sensor. Optical patterns projected onto the photodetector array and the reconstructed images: (I) square pattern; (II) letter “M;” and (III) white-light parallel strips with varying intensities. The range of the grayscale bars is from 0 (black) to 300 nA (white). (**d**) Photographs of the concentric photodetector array printed onto the inner surface of a hemispherical glass dome: (I) inside view and (II) outside view of the 3D-printed concentric photodetector array. (**e**) Current–voltage characteristics of the photodetector highlighted in (**d**). The excitation light source is a 405 nm laser of varying intensities. (**f**) Characterization of the spherical photodetector array as an image sensor. (I) the projected cross mark onto the devices (denoted by the dashed outline); (II) reconstructed cross mark pattern; (III and IV) cross mark pattern projected onto the spherical photodetector array rotated 90° and the reconstructed cross mark pattern. The range of the grayscale bar is from 0 (black) to 300 nA (white). Scale bars are 5 mm.” [Park, S.H.; Su, R.; Jeong, J.; Guo, S.-Z.; Qiu, K.; Joung, D.; Meng, F.; McAlpine, M.C. 3D Printed Polymer Photodetectors. Adv. Mater. 2018, 30, 1803980, Copyright Wiley-VCH GmbH. Reproduced with permission].

Soft optoelectronic devices printed by AM have a diverse field of application including omnidirectional light-sensing, and light-emitting which are typically used for detecting structural defects [151]. Optoelectronic structures fabricated through FDM of multi-material filaments have been shown by Loke et al. [151]. The specialized filaments were performed to contain photodetecting or light-emitting structures at their core. This allowed a good adhesion between the elements that made up the optoelectronic structures and the production of application-ready parts using a single-step process. The method for fabricating filaments was proposed for a wider range of materials and could prove to be the next evolution in the multi-material extrusion of functional devices. Another approach for building complete optoelectronic structures using AM consisted in depositing photodetectors onto flexible elastomeric substrates using a DIW method as shown in Figure 9 [148]. The photodetectors were realized through a multi-material deposition of the sensing and conductive elements, and the process was highly adaptable to various types of substrate materials both, rigid and soft, and geometries including flat and concave. The expanded flexibility of AM allowed for accurate photo sensors with new capabilities including onboard powering, as well as wearability.

Researchers and scientists have carried out significant work in the field of photonics and optoelectronic applications; however, there are still plenty of opportunities for expanding the properties, applications, and interconnectivity of optoelectronic devices. Future 3D printers’ capability to control optical properties (like refractive index, reflectivity, transmittance, absorption, diffusion, etc.) will empower flourishing design space for sensing, display, and illumination. 

## 4. Conclusions

A comprehensive review, including a brief description of AM techniques for soft functional materials, recent developments promoting their functional properties, and their use in various applications, is presented. AM technology has significantly contributed to the recent surge in the development of soft materials with functional properties such as the ability to self-heal, change color, program shape, serve as electronic devices, and multi-functionality. The versatility of AM technology has allowed for tunability of properties and greater freedom of design, boosting their potential in various applications, from tissue engineering to soft robotics. Additionally, the ability to integrate multi-materials in a single print through and flexibility in the design and fabrication of complex geometries such as lattice structures has helped to achieve high-performance integrated active electronic materials such as soft optoelectronic devices and energy harvesters. 

The lower cost of manufacturing, rapid prototyping, fabrication of complex geometry and custom building has given AM technology a competitive edge over traditional methods in the manufacturing of functional materials. However, there are a few drawbacks that may require further research and development in the technology. The drawbacks include limited printable materials, limited use of multilaterals in AM of soft devices, and in some cases low printing speed. With enormous research efforts on the AM of soft functional materials, many challenges are being overcome in this field. For example, technology such as the SLAM technique is being developed to overcome the shortcomings of AM such as low material viscosity and allowing for materials to achieve more complex designs that would otherwise not be compatible with AM. Alternatively, manipulations of the molecular structures of materials or incorporation of functional fillers into existing materials have also been shown to improve the printability of soft functional materials and expand the list of printable materials in AM technology. Additionally, incorporating an expansion of design-based software with more informed inputs based on material genomics, multiscale modeling, topology optimization, and further use of multi-material AM technology will facilitate the integration and sophistication stage in AM soft structures. The increased amount of funding and massive research and development in 3D printing technology will bring a revolution in soft functional materials. 

## Figures and Tables

**Table 1:** Summary of advantages and disadvantages of additive manufacturing methods and techniques used for the fabrication of soft functional materials.

Printing Technology	Specific Methodology	Deposition	Feature Size	Materials	Features	Drawbacks
Material Extrusion	Fuse Deposition Modeling	Line	≈200 µm [13]	Thermoplastics	Low maintenance Low cost Simplicity Potential for multi-material printing	Voids Limited to complex geometric prints
	Direct Ink Write	Line	≈120 µm [14]	Thermoplastics Thermosets Elastomers Hydrogels Nanoparticles	Large availability of materials Rapid prototyping Potential for multi-material printing	Warping Cracks Post-processing
Vat Photopolymerization	Stereolithography	Light single point	≈50 µm [15]	Thermoset Elastomers Acrylate resins Nanoparticles	High resolution Close tolerances Smooth surface finishes Complex geometric prints	Low material availability Long printing times Extensive post-processing
	Micro-Stereolithography	Light single point	≈10 µm [16]			
	Two-Photon Polymerization	Light single point	≈0.3 µm [17]			
	Continuous Liquid Interface Production	Light entire layer	≈0.4 µm [18]	Thermoplastic Acrylate resins Nanoparticles	High resolution Wavelength multiplexing	Low material availability Post-processing
	Digital Light Processing	Light entire layer	≈200 µm [19]			
Material Jetting	Drop on Demand	Drop	≈32 µm [20]	Polymers Thermoplastic Acrylate resins Elastomers Nanoparticles	High accuracy Little to no post-processing Capability to fabricate functionally graded materials Potential for multi-material printing	Inconsistent material droplet spread
	Nanoparticle Jetting	Drop	≈10 µm [21]			

**Table 2:** Summary of recent studies in the fabrication of SMPs using AM technologies and their impact on mechanical properties including shape fixity and shape recovery.

Materials	Technique	Glass Transition Temperature (°C)	Elastic Modulus (MPa)	Elongation at Break (%)	Durability (Cycles)	Shape Fixity (%)	Shape Recovery (%)	Elastic Modulus (MPa)	Elongation at Break (%)	Ref.
tBA, DEGDA with nanosilica fillers	DLP	56.23	-	85.2	10	100	90-97	-	85.2	[24]
filaflex embedded with polycaprolactone	FDM	70	48	700	10	76	97	48	700	[25]
N-butyl Acrylate	UV-assisted DIW	95.2	610	25.4 (Ultimate strain)	3	97.1	98.5	610	25.4 (Ultimate strain)	[26]
Polycyclooctene with boron nitrate and MWCNT	FDM	70	3.85 (Storage modulus)	-	-	98.9	99.2	3.85 (Storage modulus)	-	[27]
Polylactic acid (PLA)/Fe_3_O_4_ composites	FDM	66.6	1600 (Storage modulus)	-	-	96.8	96.3	1600 (Storage modulus)	-	[28]
poly(dimethyl acrylamide-costearyl acrylate and/or lauryl acrylate) (PDMAAm-co-SA)	SLA	-	-	-	3	99.8	87.6	-	-	[29]
2-Methacryloyloxy 4-formylbenzoate	DLP	57	57 ± 4.0	39.30 ± 1.0	3	97.5 ± 0.30	91.4 ± 0.20	57 ± 4.0	39.30 ± 1.0	[30]
Poly(ethylene terephthalate) (PET)	FDM	85–100	-	45	7	100	90–98	-	45	[31]
poly(ethylene glycol) dimethacrylate (PEGDMA), isobornyl acrylate and 2-ethylhexyl acrylate	DLP	125	-	-	10	92.6	95.3	-	-	[32]

**Table 3:** Summary of recent studies on AM processable self-healing polymers highlighting mechanical robustness and healing efficiency.

Materials	Tensile Strength	Max Strain	Technique	Application	Self-Healing	Stimulus	Healing Time	Efficiency	Ref.
Semi-interpenetrating polymer network elastomer	5 MPa	600%	UV-assisted DIW	Biomedical Devices	Embedded semicrystalline thermoplastic	Heat at 80 °C	20 min	<30%	[43]
Ferrogel	-	288%	DIW Bioprinting	Drug Delivery and Tissue Engineering	Reversible Imine Bond Formation	No Stimulus	10 min	~95%	[44]
Dynamic Covalent Polymer Networks	3.3 MPa	140%	FDM	-	Diels–Alder Reaction	Heat at 80 °C Deionized Water at RT	12 h	96% ~70%	[45]
Photoelastomer Ink	16 kPa	130%	SLA	Soft Actuators, Structural Composites, Architected Electronics	Disulfide Exchange	Heat at 60 °C	2 h	100%	[46]
Fluid Elastic Actuators	13–129 kPa	45–400%	SLA	Soft Robotics	Unreacted Prepolymer Resin	Sunlight ~15,000 cd m^2^	30 s	-	[47]
Physically Crosslinked Hydrogels	95 kPa	1300%	SLA	Flexible Devices, Soft Robotics, Tissue Engineering	Hydrophobic Association	Contact	6 h	~100%	[48]
Silicone Elastomer	~225 kPa	~330%	SLA	Endurable Wearables, Flexible Electronics	Ionic Bonding	Heat at 100 °C	12 h	>90%	[49]
Host–Guest Supramolecular Hydrogel	0.3–0.5 MPa	70%	DIW	Biomedical	Host–Guest Interactions	Mechanical Stress	1 h	Up to 80%	[50]
PEDOT:PSS with Polymeric Surfactant	3 MPa	35%	DIW	Energy Harvesting	Surfactant	Electrical Current 10–3 A	1 s	85% electrical output	[51]
Polyurethane Elastomer	3.39 ± 0.09 MPa	400.38%	DLP	-	Disulfide Exchange	Heat at 80 °C	12 h	95% First healing	[52]

**Table 4:** Summary of various soft electronic polymers recently manufactured through AM for use as sensors and actuators and their performances achieved.

Material	Application	Role of Printed Material	Young’s Modulus or Elasticity	Max Strain	Printing Technique	Performance	Ref.
Silicone elastomer	Dielectric actuator	Actuating layer	≈700 kPa	600%	DOD	A maximum areal strain of 6.1% at an electric field of 84.0 V/μm	[61]
Reduced graphene oxide-elastomer nanocomposites	Dielectric actuator	Flexible electrode layer	-	104%	Aerosol Jet Printing	Electrodes with a maximum stretchability of 100% could be bonded to dielectric layers without losing conductivity	[62]
Thermoplastic polyurethane/carbon nanotubes/silver nanoparticles composites	Dielectric actuator	Actuating material	3.44 MPa in print direction	Up to 800% in print direction	FDM	Dielectric constant of 6.32 and a radial extension of 4.69% at an applied 4.67 kV	[79]
Barium titanate filled silicone elastomer	Dielectric actuator	Actuating layer	39.82 kPa	>100%	DIW	Maximum tip displacement of 6 times its thickness at 5.44 kV Blocking force of 17.27 mN and deflection of 0.85 mm under a 0.12 g mass with a 5 kV applied	[63]
Thermoplastic elastomer	Dielectric actuator	Elastic frame	-	-	FDM	A tilt angle of 128° and a blocked force of 24 mN were measured when driven by 6 kV	[64]
Thermoplastic polyurethane	Dielectric actuator	Elastic frame	-	-	FDM	Honeycomb structures could achieve a longitudinal strain of 15.8% and transverse strain of −0.97% at a driving voltage of 7.5 kV	[65]
Thermoplastic polyurethane and multiwalled carbon nanotubes	Piezoresistive sensors	Sensor and electrodes	-	>100%	FDM	Anisotropic electrical resistance responses to strain with gauge factors between 1.5 and 3	[68]
Thermoplastic polyurethane and carbon nanotubes	Piezoresistive sensor	Sensor	≈1 MPa at a strain of 30%	-	FDM	Consistent 50% change in developed current during 1500 cycles of 5% strain	[67]
Thermoplastic elastomer	Piezoresistive sensor	Sensor body	-	800%	FDM	Pressure sensitivity as high as 136.8 kPa^−1^ at pressures <200 Pa and GF of 6.85 at stretching	[69]
Thermoplastic polyurethane, carbon black, and silver composites	Piezoresistive sensor	Substrate, sensor, and electrode layers	-	600% for TPU, 120% for electrode layer	DIW	Low sensitivity to in-plane stretching of (R/R_0_ < 7%) and pressure sensitivity of 5.54 kPa^−1^ at low pressures (<10 kPa)	[70]
PDMS and multi-walled CNT	Piezoresistive sensor	Conductive pattern	-	>70%	DIW	GF of 13.01 with linear responses up to 70% tensile strain	[71]
Ionogels	Piezoresistive sensor	Sensor	Tensile strength of 0.81 MPa at 242% strain	242%	DIW	≈40% change in electrical current under a 29% elongation	[72]
Cellulose nanocrystals and deep eutectic solvent ionogel	Piezoresistive sensor	Sensor	0.20 MPa	At least 790%	DIW	Up to a 3.3 GF in the strain range up to 300% strain	[73]
Barium titanate	Piezoelectric sensor	Sensing lattice	-	-	Ceramic slurry DLP	Compressibility up to at least 10% and direct relationship between recorded voltage and applied strain	[78]
Polyvinylidene fluoride and sodium potassium niobate	Piezoelectric sensor	Sensor	<1.0 MPa for designed structure	40%	FDM	Pressure sensitivity of 2.295 mV kPa^−1^ and ability to self-power	[76]
Lead Zirconate Titanate	Piezoelectric sensor	Sensor	Compliance up to 3 × 10^−8^ Pa	-	Ceramic slurry DLP	Variable piezoelectric charge constant up to 110 pC/N and sensitivity to pressure and extension with high conformability to surfaces	[77]

**Table 5:** Summary of chromic materials and their properties that have been recently processed through AM techniques.

Materials	Matrix	Response Type	Technique	Applied Stimuli	Color-Change	Absorbance Wavelength (nm)	Reversibility	Ref.
Spiropyran	Polydimethylsiloxane	Mechanochromic	DIW		Off-white to Purple		Yes	[80]
Poly(butyl acrylate)	Polyacrylamide	Mechanochromic and Hydrochromic	DIW	Compression: 5.7 kPa	Entire Wavelength Spectrum Colors	500–900	Yes	[81]
Polyethyleneimine-co-poly(acrylic acid)	Polyethylene glycol diacrylate	Hydrochromic	DIW		Blue-Green, and Red	400–650	Yes	[81]
Polyurethane acrylate	Isobornyl acrylate	Thermochromic	Projection Micro Stereolithography	74.2–81.7 °C	Black, Red, Blue, Yellow, White	400–800	Yes	[82]
Poly(N-isopropylacrylamide)	Silica-alumina based gel	Thermochromic and Electrochromic	DIW	>60 °C and 0.6–1.8 Amp (2~6 V)	Transparent to Opaque State	1400–1900	Yes	[83]
Poly(3,4 ethylenedioxythiophene)-poly(styrene sulfonate) (PEDOT:PSS)/silver (Ag)	Grid/polyethylene terephthalate	Electrochromic	IJ	−0.6–0 V	Transparent, Black	633	Yes	[84]

**Table 6:** Different multifunctional soft materials recently manufactured using AM techniques and their applications.

Materials	Modulus	Max Strain (%)	Technique	Application	Functionalities	Ref.
Polyacrylamide	17 KPa	574	DIW	Biocompatible Soft Robotics	Magnetic response	[91]
Polyacrylamide with Carbomer	40 KPa	260	DIW	Biocompatible Soft Robotics	Magnetic response	[91]
PLA-PEA	125 MPa	2.5	DIW	Actuation and Sensing	Shape memory effect and piezoelectric effect	[92]
Polypyrrole (PPy)	498 kPa	1500	DIW	Sensor	Self-healing	[93]
Polydimethylsiloxane (PDMS)	160 kPa	210	DIW	Sensor	Superhydrophobicity	[94]

## Data Availability

Not applicable.
